# Proteinaceous effector discovery and characterization in filamentous plant pathogens

**DOI:** 10.1111/mpp.12980

**Published:** 2020-08-07

**Authors:** Claire Kanja, Kim E. Hammond‐Kosack

**Affiliations:** ^1^ Department of Biointeractions and Crop Protection Rothamsted Research Harpenden UK; ^2^ School of Biosciences University of Nottingham Nottingham UK

**Keywords:** bioinformatic effector predictions, effector host–target interactions, effectors, fungal phytopathogens, in planta methodologies, oomycete phytopathogens

## Abstract

The complicated interplay of plant–pathogen interactions occurs on multiple levels as pathogens evolve to constantly evade the immune responses of their hosts. Many economically important crops fall victim to filamentous pathogens that produce small proteins called effectors to manipulate the host and aid infection/colonization. Understanding the effector repertoires of pathogens is facilitating an increased understanding of the molecular mechanisms underlying virulence as well as guiding the development of disease control strategies. The purpose of this review is to give a chronological perspective on the evolution of the methodologies used in effector discovery from physical isolation and in silico predictions, to functional characterization of the effectors of filamentous plant pathogens and identification of their host targets.

## Graphical abstract

The chronological progression of methodologies used in effector discovery; from physical isolation and in silico predictions, to functional characterization and host target identification.

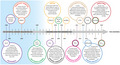

## 1 INTRODUCTION

If people think Nature is their friend, then they sure don't need an enemy.

Kurt Vonnegut, *Letter in* Time *magazine*

### 1.1 The threats from filamentous phytopathogens

Our expanding global population forces us to intensify our crop production as we prepare to feed 2.2 billion more people by 2050. One of the main biotic challenges facing society to meeting these ever‐growing demands are filamentous plant pathogens. Oomycetes and fungi are the causal agents of some of the most notorious plant diseases and are a true threat to our global food security and community structures. Plant disease outbreaks have occurred throughout human history, some of the most infamous include the Irish potato famine caused by the oomycete *Phytophthora infestans* (Turner, 2005), Panama disease caused by *Fusarium oxysporum* f. sp. *cubense* (Gordon, 2017), and wheat stem rust caused by *Puccinia graminis* f. sp. *tritici* (Roelfs, 1985; Singh *et al*., 2011).

### 1.2 Effectors and the plant immune response

The elegantly described “zig‐zag” model by Jones and Dangl (2006) reveals a two‐tier immune response where pathogen‐associated molecular patterns (PAMPs) are first detected on host cell surfaces by pattern recognition receptors (PRRs), inducing pattern‐triggered immunity (PTI). To evade this response, pathogens secrete effector proteins that manipulate the host and aid colonization, yet in hosts that have the corresponding resistance (*R*) genes (Flor, 1971), these effectors are detected by receptors such as the intracellular Nod‐like receptors (NLRs) that induce effector‐triggered immunty (ETI), resulting in a hypersenstive response (HR) and programmed cell death (de Wit, 2016, Zhang *et al*., 2017).

Just as with all models, the story is more complicated and not all features of the plant–microbe interactions are accommodated. Effectors can be highly conserved, thus not under selective pressure to evade host detection, such as the members of the oomycete Crinkler (CRN) effector family or the core fungal effector NIS1 (Depotter and Doehlemann, 2019; Irieda *et al*., 2019) whilst other effectors are detected extracellularly (van der Burgh and Joosten, 2019).

Recent studies suggest that, rather than a two‐tier system of immunity, ETI and PTI activate different but interacting pathways leading to plant immunity. The activation of the paired *Arabidopsis* NLRs RRS1‐R and RPS4 by the bacterial effector AvrRps4 cannot induce HR without the presence of PAMPs (Ngou *et al*., 2020). Both co‐ and predelivery of AvrRps4 with PAMPs leads to an increased and prolonged expression of PTI‐associated defence genes such as *BIK1*, *BAK1*, and *Rboh*; the expression of these genes is not induced by effectors alone (Ngou *et al*., 2020). Similarly, ETI responses in *Arabidopsis* mutants lacking PRRs are greatly compromised, with the ETI‐induced reactive oxygen species (ROS) production being mediated by PRRs (Yuan *et al*., 2020). This suggests that PTI is a required component of ETI with mutual potentiation of immune mechanisms triggered by intracellular and cell‐surface receptors.

### 1.3 The importance of effector research

Hundreds of small proteins, predicted to be effectors, are secreted by filamentous phytopathogens during host colonization (Dean *et al*., 2005; Kämper *et al*., 2006; Yoshida *et al*., 2009; Duplessis *et al*., 2011). We have little understanding of the function of most of these putative effectors and each typically shares minimal or no sequence homology to proteins with previously defined functions. However, the effector repertoire of a pathogen is a major determinant of host specialization and can greatly impact whether the plant–pathogen interaction is successful or not based on the genotype of the host (Raffaele *et al*., 2010; Sánchez‐Vallet *et al*., 2018a)

Molecular studies have characterized over 60 fungal effectors across multiple species; however, this barely makes a dent in the candidate effector repertoire for each pathogenic species (Sperschneider *et al*., 2015). For example, the barley powdery mildew fungus *Blumeria graminis* f. sp. *hordei* alone is suspected to have roughly 7% of its genome encoding candidate secreted effector proteins (CSEPs) (Pedersen *et al*., 2012).

Identifying and characterizing the function of effector proteins will improve our understanding of their role in disease formation and influence our future strategies to combat pathogen infections. Fundamental effector research is a key part of devising new plant disease control strategies and this is detailed further in Sections 3.2 and 6 of this review. Effectors play an important role in crop breeding where, as well as being used to detect resistance genes in new cultivars, characterized effectors can be used to locate susceptibility loci in vulnerable crops (Vleeshouwers and Oliver, 2014). The development of mobile sequencing technology means that genes encoding effectors can also be used to detect the emergence of new strains of crop pathogens in the field and elude the severity of future disease outbreaks (Radhakrishnan *et al*., 2019). Effectors function in multiple ways, including inhibiting host enzymes, modulating plant immune responses, and targeting host gene‐silencing mechanisms. All features of effectors described in this article are summarized in Table 1, including their mode of action where known.

**TABLE 1:** List of filamentous phytopathogen species and their effectors referred to in this review

Effector	Size aa^a^	Uniprot ID	Biological function	Species	Disease name	Host	Reference
ATR13	187	M4C367	Secreted effector that acts as an elicitor of the HR specifically on plants carrying the defence protein RPP13	*Hyaloperonospora parasitica*	*Arabidopsis* downy mildew	*Arabidopsis thaliana*	Sohn *et al. * (2007)
ATR1^NDWsB^	311	M4B6G6	Secreted effector that acts as an elicitor of the HR specifically on plants carrying the cognate R defence protein RPP13	*H. parasitica*	*Arabidopsis* downy mildew	*A. thaliana*	Sohn *et al. * (2007)
Avr1b‐1	204	G5A9E5	Uncharacterized	*Phytophthora sojae*	Stem and root rot of soybean	Soybean (*Glycine max*)	Shan *et al. * (2004); Dou *et al*. (2008)
			Has been shown to reduce heterologously induced plant cell death				
Avr1‐C039	89	NA	Uncharacterized protein that is recognized in the host by direct binding of the NB‐LRR proteins RGA5, which together with RGA4 induces ETI	*Magnaporthe oryzae*	Rice blast	Rice (*Oryza sativa*)	Farman and Leong (1998) ; Cesari *et al*. (2013); Ribot *et al. * (2013)
Avr2	78	Q8NID8	Inhibits several apoplastic Cys proteases, including the tomato protease Rcr3, which is required for plant basal defence and induces HR in tomato races that carry the cognate R protein Cf‐2	*Cladosporium fulvum*	Tomato leaf mould	Tomato (*Solanum lycopersicum*)	Rooney *et al*. (2005); van Esse *et al*. (2008); Song *et al. * (2009)
Avr3a	147	E2DWQ7	Suppresses host BAK1/SERK3‐mediated immunity, by targeting and stabilizing host E3 ligase CMPG1	*Phytophthora infestans*	Potato late blight	Potato (*Solanum tuberosum*)	Armstrong *et al*. (2005)
Avr4	135	Q00363	Chitin binding lectin, which inhibits plant chitinases to minimize chitin hydrolysis and also induces HR in tomato races that carry the cognate R protein Cf‐4	*C. fulvum*	Tomato leaf mould	Tomato (*S. lycopersicum*)	Joosten *et al*. (1997); van den Burg *et al. * (2006)
Avr9	63	P22287	Induces necrosis by triggering HR in tomato containing the cognate R protein Cf‐9	*C. fulvum*	Tomato leaf mould	Tomato (*S. lycopersicum*)	De Wit *et al*. (1985)
AVR_a9_	102	N1J9M0	Uncharacterized but is recognized by the intracellular MLA10 receptor in barley and results in HR	*Blumeria graminis* f. sp. *hordei*	Barley powdery mildew	Barley (*Hordeum vulgare*)	Saur *et al. * (2019a)
AvrL567‐A	150	Q6R661	Triggers resistance responses in flax containing the cognate R proteins L5 and L6	*Melampsora lini*	Flax rust	Flax (*Linum usitatissimum*)	Wang *et al.* (2007)
AvrL567‐D	150	Q1HBK6	Triggers resistance responses in flax containing the cognate R protein L6	*M. lini*	Flax rust	Flax (*L. usitatissimum*)	Wang *et al.* (2007)
AvrLm1	205	Q258K5	Interacts with the host protein (MAP) kinase 9 (BnMPK9), causing self‐increased protein accumulation and enhanced phosphorylation, resulting in the induction of cell death	*Leptosphaeria maculans*	Blackleg	Oilseed rape (*Brassica napus*)	Soyer *et al.* (2014); Fouché *et al*. (2018); Ma *et al. * (2018b)
Avr‐Pik and Avr‐PikD	113	C4B8C1	Induces HR in rice races containing the corresponding cognate R protein *Pik*	*M. oryzae*	Rice blast	Rice (*O. sativa*)	Li *et al*. (2019); Yoshida *et al*. (2009)
			AvrPikD is a novel allele of Avr‐Pik				
AvrSr35	578	A0A2I6B3G6	Uncharacterized but interacts with the Sr35 immune receptor	*Puccina graminis* f. sp. *tritici*	Wheat stem rust	Wheat (*Triticum* sp.)	Salcedo *et al*. (2017)
AvrStb6	86	A0A2K9YW36	Uncharacterized, induces HR in wheat cultivars containing the cognate R protein Stb6	*Zymoseptoria tritici*	Septoria leaf blotch	Wheat (*Triticum* sp.)	Zhong *et al*. (2017)
BAS1	115	G5EHI7	Induces an early, basal defence response such as ROS production and callose deposition in susceptible rice	*M. oryzae*	Rice blast	Rice (*O. sativa*)	Yang *et al. * (2017)
BEC1011 and BEC1054	118	N1JJX4	Noncatalytic homologue of fungal RNase that competitively binds host RNA to inhibit the degradation of the ribosomal RNA by RIPs, preventing host cell death	*B. graminis* f. sp. *hordei*	Barley powdery mildew	Barley (*H. vulgare*)	Pennington *et al*. (2019)
		N1JJ94					
Capsicein	98	P15571	Induces incompatible HR Elicits leaf necrosis and causes the accumulation of pathogenesis‐related proteins	*Phytophthora capsici*	Stem and fruit rot	Capsicum (*Capsicum annuum*)	Ricci *et al. * (1989)
Cce1	129	A0A0D1C5E3	Uncharacterized but may inhibit early PTI response in planta	*Ustilago maydis*	Corn smut	Maize (*Zea mays*)	Seitner *et al*. (2018)
Cinnamomin	98	P15569	Induces incompatible HR	*Phytophthora cinnamomi*	Phytophthora root rot	>4,000 species including cinnamon (*Cinnamomum verum*)	Huet and Pernollet (1989)
			Elicits leaf necrosis and causes the accumulation of pathogenesis‐related proteins				
Cmu1	290	A0A0D1DWQ2	Interferes with the activity of host cytosolic chorismate mutase and inhibits the biosynthesis of salicylic acid required for plant defence signalling	*U. maydis*	Corn smut	Maize (*Z. mays*)	Djamei *et al*. (2011)
Cryptogein	118	P15570	Induces incompatible HR	*Phytophthora cryptogea*	Tomato foot rot	Tomato (*S. lycopersicum*)	Ricci *et al. * (1989)
			Elicits leaf necrosis and causes the accumulation of pathogenesis‐related proteins				
Ecp1	96	Q00364	Extracellular protein that triggers Cf‐Ecp1 mediated resistance	*C. fulvum*	Tomato leaf mould	Tomato (*S. lycopersicum*)	Laugé *et al*. (1997)
Ecp2	165	Q00365	Extracellular protein that triggers Cf‐Ecp2 mediated resistance	*C. fulvum*	Tomato leaf mould	Tomato (*S. lycopersicum*)	Laugé *et al*. (1997)
Ecp6	222	A0A1P8YXP5	Ecp6 contains LysM domains, which bind to the fungal cell wall chitin with ultra‐high affinity, preventing detection by the host PRRs	*C. fulvum*	Tomato leaf mould	Tomato (*S. lycopersicum*)	De Jonge *et al*. (2010); Sánchez‐Vallet *et al.* (2013)
EPIC1 and EPIC2	126	A1L015	Inhibits several apoplastic Cys proteases, including the tomato protease Rcr3, which is required for plant basal defence	*P. infestans*	Potato late blight	Potato (*S. tuberosum*)	Song *et al*., 2009); Tian *et al*. (2007)
	125	A1L017					
INF1	118	Q01905	A PAMP elicitor of plant cell death that targets the receptor kinase BAK1	*P. infestans*	Potato late blight	Potato (*S. tuberosum*)	Kamoun *et al*. (1997); Chaparro‐Garcia *et al*. (2011)
MLP124266 and MLP1124499	69	NA	Uncharacterized	*Melampsora larici‐populina*	Poplar rust	Poplar (*Populus* sp.)	de Guillen *et al*. (2019)
	50						
MoCDIP4	294	G4MVX4	Induces cell death in rice protoplasts	*M. oryzae*	Rice blast	Rice (*O. sativa*)	Chen *et al. * (2012)
NIS1	162	N4VG36	Targets the immune kinases BAK1 and BIK1 and disrupts downstream PTI responses	*Colletotrichum orbiculare*	Cucumber anthracnose	Cucumber (*Cucumis sativus*)	Yoshino *et al*. (2012); Irieda *et al.* (2019)
Para1	118	P41801	Induces incompatible HR in plants from the Solanaceae and Brassicaceae families	*Phytophthora parasitica*	Potato buckeye rot	Potato (*S. tuberosum*)	Kamoun *et al*. (1993)
			Elicits leaf necrosis and causes the accumulation of pathogenesis‐related proteins				
Pep1	178	G0X7E8	Inhibition of the plant oxidative burst by directly interacting with the peroxidase POX12, which generates ROS as a PTI response	*U. maydis*	Corn smut	Maize (*Z. mays*)	Doehlemann *et al*. (2009)
PiAvr2	116	A0A2D1N523	An RxLR effector that induces HR when interacting with the host NB‐LRR protein R2	*P. infestans*	Potato late blight	Potato (*S. tuberosum*)	Saunders *et al*. (2012a)
Pit2	120	A0A0D1EAR7	Modulates host immunity by acting as a substrate mimic for apoplastic maize PLCPs, including CP1A, CP1B, XCP2, and CP2 processing of Pit2 releases the embedded inhibitor peptide PID14, which in turn blocks PLCP activity	*U. maydis*	Corn smut	Maize (*Z. mays*)	Mueller *et al*. (2013)
PsIsc1	210	NA	An isochorismatase that supresses the precursor to the plant salicylate metabolism pathway and the subsequent salicylate‐mediated defences in planta	*P. sojae*	Stem and root rot of soybean	Soybean (*G. max*)	Liu *et al. * (2014)
PSTha5a23	108	NA	Uncharacterized but is involved in PTI suppression	*P. graminis* f. sp. *tritici*	Wheat stem rust	Wheat (*Triticum* sp.)	Cheng *et al*. (2017)
PWL2	145	A0A3G2LZW6	Avirulence proteins in interactions involving weeping lovegrass and finger millet	*M. oryzae*	Rice blast	Rice (*O. sativa*)	Khang *et al*. (2010)
Rsp3	869	A0A0D1DYI3	Required for anthocyanin accumulation and blocks the antifungal activity of mannose‐binding maize proteins AFP1 and AFP2	*U. maydis*	Corn smut	Maize (*Z. mays*)	Ma *et al. * (2018a); Seitner *et al*. (2018)
Six1/Avr3	154	M1GN93	Induces necrosis by triggering HR in tomato containing the cognate R protein I‐3	*Fusarium oxysporum* f. sp. *lycopersici*	Tomato wilt	Tomato (*S. lycopersicum*)	Rep *et al. * (2004)
Six3/Avr2	163	D0U2D2	Avr2 suppresses PTI responses, such as growth inhibition, ROS production, MAPK activation, and callose deposition	*F. oxysporum* f. sp. *lycopersici*	Tomato wilt	Tomato (*S. lycopersicum*)	Houterman *et al*. (2009); Di *et al. * (2017)
Tin2	207	NA	Masks a ubiquitin‐proteasome degradation motif in ZmTTK1 thereby stabilizing the anthocyanin biosynthesis pathway and decreases levels of metabolites available for plant defences	*U. maydis*	Corn smut	Maize (*Z. mays*)	Tanaka *et al*. (2014)
ToxA	178	P78737	Proteinaceous toxin that causes necrotic lesions on infected leaves	*Pyrenophora tritici‐repentis*	Tan spot	Wheat (*Triticum* sp.)	Tomas *et al*. (1990); Ballance *et al*. (1996); Ciuffetti *et al*. (1997); Welti and Wang (2004)
Vd2LysM	145	G2X4U8	Uncharacterized	*Verticillium dahliae*	Verticillium wilt	Tomato (*S. lycopersicum*)	de Jonge *et al*. (2013)
VdIsc1	190	NA	An isochorismatase that supresses the precursor to the plant salicylate metabolism pathway and the subsequent salicylate‐mediated defences in planta	*V. dahliae*	Verticillium wilt	Multiple species	Liu *et al. * (2014)

Abbreviations: HR, hypersensitive response; NB‐LRR, nucleotide‐binding domain (NB) and a leucine‐rich repeat (LRR); PAMP, pathogen‐associated molecular pattern; PLCP, papain‐like cysteine protease; PRR, pattern recognition receptor; PTI, pattern‐triggered immunity’ RIP, ribosome‐inactivating protein; ROS, reactive oxygen species.

^a^ Number of amino acids including signal peptide.

## 2 THE CHRONOLOGICAL PERSPECTIVE OF FINDING EFFECTORS

There is nothing like looking, if you want to find something.

J. R. R. Tolkien, *The Hobbit, or There and Back Again*

### 2.1 The proteomics approach

Some of the best‐characterized effector proteins come from the biotrophic fungal pathogen *Cladosporium fulvum*, the causal agent of tomato leaf mould and an early model system for fungal effector discovery. *C. fulvum* avirulence (Avr) effectors are a classic example of the gene‐for‐gene model. The detection of the Avr effector by the host carrying the cognate *R* gene can induce a strong immune response in the plant and inhibit *C. fulvum* colonization (Flor, 1971; De Wit *et al*., 1986).

**FIGURE 1:**
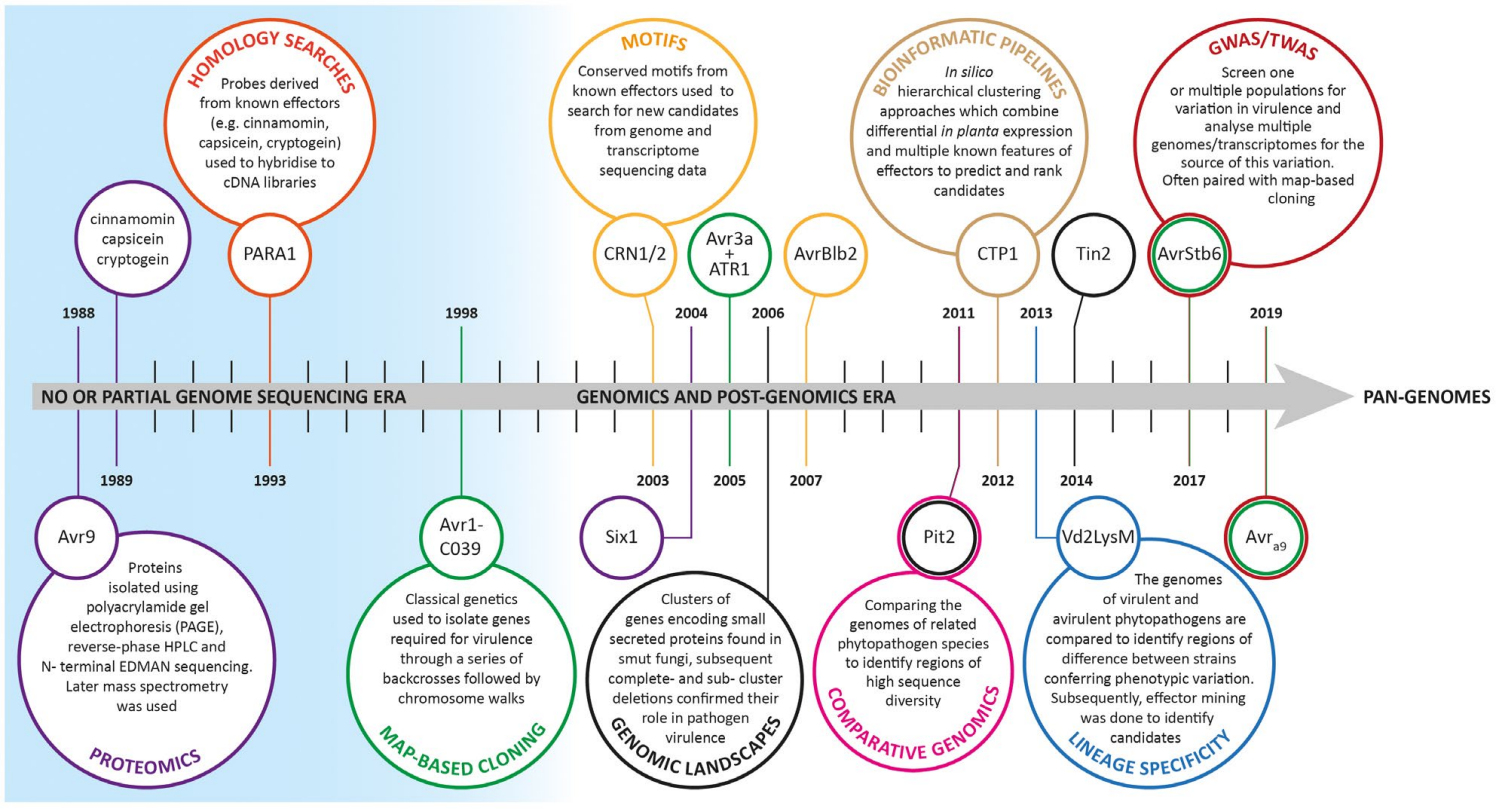
A timeline showing the progression of filamentous plant pathogen effector prediction and identification from the pregenomic era to the present day. The first effectors identified using these methods are included as well as the elicitins used for homology‐based searches. Increasingly, pangenome data are used to predict core and novel candidates but as yet none have been characterized using this technique. For a recent review of pangenomics see Golicz *et al*. (2019). Details on individual effectors named are given in Table 1.

Early in planta studies took advantage of the fact that *C. fulvum* only colonizes the tomato leaf apoplast. Secreted proteins could be isolated by collecting apoplastic wash fluid from *C. fulvum‐*infected tomato leaves and studying the effects of this fluid on a range of tomato varieties (De Wit *et al*., 1985). When fluid collected from plants infected with *C. fulvum* races harbouring the *avr9* gene was infiltrated into the near‐isogenic tomato leaves carrying the *Cf‐9* gene a strong HR was triggered. Treating this fluid with proteases confirmed the Cf‐9‐mediated HR was triggered by proteinaceous entities (De Wit *et al*., 1986). The subsequent purification of the small Avr9 (Figure 1) then led to the first fungal *Avr* gene to be cloned, whilst its low expression profile in vitro suggested for the first time that the host plant plays an important role in inducing *Avr* expression (Schottens‐Toma and de Wit, 1988; van Kan *et al*., 1991; Van den Ackerveken *et al*., 1992, 1994). The mature Avr9 is a 28 amino acid protein with a high percentage of cysteines (*n* = 6), features that become important in many subsequent effector identification stories (van Kan *et al*., 1991).

This apoplastic proteomics approach was successfully used to identify additional small cysteine‐rich *C. fulvum* effectors such as Avr4 (Schottens‐Toma and de Wit, 1988; van den Burg *et al*., 2006) and was employed to identify Six1 (Avr3) and Six3 (Avr2) in *Fusarium oxysporum* f. sp. *lycopersici* (Fol) (Rep *et al*., 2004; Houterman *et al*., 2007; Houterman *et al*., 2009).

### 2.2 Homology searches

Once an effector has been cloned, the sequence can be used to identify homologous candidates in closely related species. Three elicitins were isolated from *Phytophthora* spp. using proteomics techniques: cryptogein (*P. cryptogea*), cinnamomin (*P. cinnamomi*), and capsicein (*P. capsici*) (Huet and Pernollet, 1989, Ricci *et al*., 1989). Primers were deigned based on conserved regions of the elicitin amino acid sequences and used to probe cDNA libraries from *P. parasitica*, leading to the discovery of the host‐specific elicitor protein PARA1 (Kamoun *et al*., 1993).

### 2.3 Genetic mapping

Prior to the genomics era, the isolation of Avr proteins from intracellular colonizing fungal pathogens such as *Magnaporthe oryzae* and haustoria‐producing pathogens was unsuccessful using the proteomics approach. Instead, in the case of the rice blast fungus *M. oryzae*, map‐based cloning techniques were used to clone Avrs such as Avr1‐CO39 (Farman and Leong, 1998). Avr1‐CO39 was mapped to a region on chromosome 1 by a series of backcrosses of the progeny of the virulent isolate Guy11 and the avirulent isolate 2539 (Smith and Leong, 1994). Later, a chromosome‐walking strategy led to the physical mapping and identification of Avr1‐CO39. The identity of the Avr1‐CO39 locus was confirmed by transforming the virulent Guy11 strain with cosmids from the Avr1‐CO39 genetic interval. This resulted in a loss of pathogenicity on rice cultivars containing the corresponding functional CO39 resistance gene (Farman and Leong, 1998).

### 2.4 Always lagging behind

By the end of the 20th century, over 30 bacterial *Avr* genes had been cloned and characterized by screening cosmid libraries, with almost all of these coming from two host‐specific species of *Pseudomonas* and *Xanthomonas* (Leach and White, 1996; De Wit, 1997). In comparison, using proteomics and genetic mapping, only eight fungal phytopathogen Avr genes had been successfully identified and confirmed to be effectors (Laugé and De Wit, 1998). But all this was about to change.

### 2.5 Sanger and next‐generation sequencing of pathogen genomes

In the early 2000s, the Fungal Genome Initiative (FGI) was established following the publication of a white paper (Birren *et al*., 2003) to promote the sequencing in the public domain of fungal genomes belonging to species important to human health, agriculture, and industry. By 2017 a total of 191 genomes of fungal plant pathogens had been sequenced, including the economically important *M. oryzae, Fusarium graminearum*, and *Botrytis cinerea* (Dean *et al*., 2005, 2012; Cuomo *et al*., 2007; Amselem *et al*., 2011; Aylward *et al*., 2017). This, together with the publication of numerous oomycete genomes, including the late potato blight pathogen *Phytophthora infestans* (Haas *et al*., 2009), as well as extensive in planta and in vitro transcriptome data sets, has led to an explosion in effector discovery. These techniques for effector discovery are summarized in Table 2.

**TABLE 2:** Approaches and techniques deployed for effector discovery and the initial proteins/genes successfully isolated

Technique	Effector	Species	Reference
Proteomics	Avr9	*Cladosporium fulvum*	Schottens‐Toma and de Wit (1988); van Kan et al. (1991)
	Six1	*Fusarium oxysporum* f. sp. *lycopersici*	Rep *et al*. (2004)
Map‐based cloning	Avr1‐CO39	*Magnaporthe grisea*	Farman and Leong (1998)
	Avr3a	*Phytophthora infestans*	Armstrong *et al*. (2005)
	ATR1	*Hyaloperonospora parasitica*	Rehmany *et al*. (2005)
Homology searches	PARA1	*Phytophthora parasitica*	Kamoun *et al*. (1993)
	INF1	*P. infestans*	Kamoun *et al*. (1997)
Motifs/secretion peptides	Crn1 and Crn2	*P. infestans*	Torto *et al*. (2003)
	AvrBlb2	*P. infestans*	Win *et al. * (2007); Oh et al. (2009)
Genomic landscapes	Tin2	*Ustilago maydis*	Kämper *et al*. (2006); Brefort *et al*. (2014)
Comparative genomics	Pit2	*U. maydis*	Doehlemann *et al*. (2011)
Bespoke bioinformatic pipelines	CTP1	*Melampsora larici‐populina*	Saunders *et al*. (2012b); Petre *et al*. (2015)
Lineage‐specific	Vd2LysM	*Verticillium dahliae*	de Jonge *et al*. (2013); Kombrink *et al*. (2017)
GWAS/TWAS	AvrStb6	*Zymoseptoria tritici*	Zhong *et al*. (2017)
	Avr_a9_	*Blumeria graminis* f. sp. *hordei*	Saur *et al*. (2019a)

## 3 REFINING EFFECTOR PREDICTION

Truth, like gold, is to be obtained not by its growth, but by washing away from it all that is not gold.

Leo Tolstoy, *Diaries*

### 3.1 Secretion

As the de Wit *et al*. studies demonstrated, a key feature of effectors is secretion by the pathogen into the host (De Wit *et al*., 1985; Asai and Shirasu, 2015). Therefore, early studies in effector discovery using sequencing data focused on the predicted secretome.

In a bid to identify extracellular effector proteins, Torto *et al*. (2003) used their PEX‐finder algorithm to mine transcript datasets of the potato pathogen *P. infestans*. The algorithm searched for a specific amino acid sequence known as a signal peptide followed by a cleavage site commonly found at the N‐terminus of secreted proteins (Nielsen and Krogh, 1998; Torto *et al*., 2003). Of the 261 cDNAs predicted to code for secreted proteins, 78 had no matches to those found in the public databases, a feature common to candidate effectors. Using high‐throughput functional expression assays this study led to the discovery of a large complex family of effectors called crinklers (CRNs), which are found throughout the pathogenic oomycetes (Schornack *et al*., 2010; Amaro *et al*., 2017).

However, some characterized secreted effectors lack a signal peptide. For example, the effectors, PsIsc1 and VdIsc1, produced by *Phytophthora sojae* and *Verticillium dahliae,* respectively, have been shown to be unconventionally secreted into the respective host to suppress salicylate (SA)‐mediated defences in planta (Liu *et al*., 2014).

Another difficulty is that such broad criteria leaves a large pool of possible effector candidates that are demanding in both time and resources to functionally characterize, with studies often having low discovery rates. The *Magnaporthe grisea* effector MC69, essential for appressoria formation (Motaung *et al*., 2017), was the only candidate from 1,306 putative secreted proteins that was found to be required for pathogenicity following large‐scale gene disruptions (Yoshida *et al*., 2009; Saitoh *et al*., 2012).

### 3.2 Domains

The *C. fulvum* effector Ecp6 sequesters the fungal cell wall protein chitin, preventing chitin fragment detection by the host PRRs, and thereby evades a host immune response (De Jonge *et al*., 2010). Ecp6 contains LysM domains that bind to chitin with ultrahigh affinity, therefore outcompeting host immune receptors (Sánchez‐Vallet *et al*., 2013). The LysM domain found in Ecp6 has now been identified in over 302 putative effectors from 62 published fungal genomes, and is conserved among effectors targeting the chitin detection aspect of plant immunity (De Jonge and Thomma, 2009; Lee *et al*., 2014).

On the other hand, the Avr2 effector from *C. fulvum* and the EPIC1 and EPIC2 effectors from *P. infestans* both target the tomato defence protease Rcr3 (Song *et al*., 2009) yet are unrelated and share no sequence similarity, thus relying on the presence of conserved domains could cause many possible candidates to be overlooked.

### 3.3 Motifs

The first four oomycete Avr effectors cloned, ATR13 and ATR1^NDWsB^ from the downy mildew *Hyaloperonospora parasitica* (Allen *et al*., 2004; Rehmany *et al*., 2005), Avr3a from *P. infestans* (Armstrong *et al*., 2005), and Avr1b‐1 from *P. sojae* (Shan *et al*., 2004), showed no sequence similarity except for two conserved motifs at the N‐terminus. These RxLR and DEER motifs have since been identified as N‐terminal host targeting domains and, in *P. infestans*, the RxLR motif in the Avr3a effector is required for translocation into potato cells (Whisson *et al*., 2007; Bos *et al*., 2010).

RxLR effectors have been identified in multiple *Phytophthora*, *Albugo*, and *Hyaloperonospora* species, with 568 RxLR genes being found in *P. infestans* alone, making this the largest oomycete effector family to date (Anderson *et al*., 2015). Rapid variation and host specialization are attributed to the general lack of sequence similarity in filamentous pathogen effectors, yet this mostly contributes to the variation in the C‐terminus of oomycete effector sequences, leaving the N‐terminal motifs largely conserved (Win *et al*., 2007). Conserved motifs such as RxLR and the more downstream DEER are used as powerful bioinformatic tools to isolate putative effector repertoires from genomic sequences (Jiang *et al*., 2008; Raffaele and Kamoun, 2012).

Within pathogenic fungi there is limited evidence for conserved translocation motifs. One possible exception is the [YFC]xC motif found in *Blumeria graminis* f. sp. *hordei* and *Puccinia* spp., members of the phyla Ascomycota and Basidiomycota, respectively (Godfrey *et al*., 2010; Duplessis *et al*., 2011). The evolutionary distance between these two fungi suggests a deep homology in the conservation of this motif, linked to a biotrophic lifestyle that uses haustoria‐based feeding.

However, the general lack of sequence similarity or conserved domains means that bioinformatic approaches to effector prediction need to go beyond sequence homology.

### 3.4 Structure

The structural properties of proteins are more highly conserved than amino acid sequences (Illergård *et al*., 2009) and therefore could be used as a tool for effector prediction. The structural similarities between the two sequenced *M. oryzae* effectors Avr1‐CO39 and Avr‐Pia were found using two‐ and three‐dimensional nuclear magnetic resonance (NMR) experiments (de Guillen *et al*., 2015) and led to the discovery of the *Magnaporthe* Avr and ToxB‐like effector family (MAX), which contains half of all cloned *M. oryzae* Avrs despite sharing less than 25% sequence identity (de Guillen *et al*., 2015).

The structural analysis of four RxLR oomycete effectors showed the presence of a conserved C‐terminus 3‐α‐helix fold (Boutemy *et al*., 2011; Yaeno *et al*., 2011). This WY domain, named after the interacting tryptophan and tyrosine residues, hints to a core, stable protein scaffold as a source of protein function (Wirthmueller *et al*., 2013).

Resolving the structure of known effector proteins provides a useful tool for supporting the candidacy of putative effectors. One of the early effectors to be structurally resolved was ToxA produced by the tan spot fungus, *Pyrenophora tritici‐repentis*. The ToxA crystal structure was resolved using X‐ray crystallography (1.65 Å) and revealed a novel β‐sandwich fold (Sarma *et al*., 2005). Later, the resolution of the flax rust, *Melampsora lini,* effectors AvrL567‐A and ‐D showed a similar β‐sandwich fold hinting at the structural homology of unrelated effector proteins (Wang *et al*., 2007).

Recently the structures of two candidate effectors in the poplar rust fungus*, Melampsora larici‐populina,* were resolved using NMR. One, MLP124266, is the first fungal protein to present a knottin‐like structure (Postic *et al*., 2017) whilst the other, MLP1124499, shares structural similarity with members of the Nuclear Transport Factor‐2 (NTF2) superfamily. In both cases these candidate effectors show no sequence homology with structurally similar proteins and are the first examples of effectors with these structures (de Guillen *et al*., 2019).

### 3.5 Rich in cysteines but not in size

The additional criteria for candidate effector selection often require secreted proteins to be small and cysteine‐rich (Sperschneider *et al*., 2015). The presence of multiple cysteines enables the formation of stabilizing disulphide bridges (De Wit *et al*., 1986; Doehlemann *et al*., 2009).

Relying on such broad criteria can be problematic as, despite many known effectors sharing these features, these are not universal requirements. NIS1, first described in the cucumber anthracnose fungus *Colletotrichum orbiculare* (Yoshino *et al*., 2012), is conserved across both Basidiomycota and Ascomycota (Irieda *et al*., 2019), but contains no cysteines.

Relying on the size of mature peptides as a parameter for effector identification can also be problematic. The maximum size of a small protein in effector discovery can be anything from 150 to 400 amino acids (Bowen *et al*., 2009; Saunders *et al*., 2012b). However, even the larger size limits would exclude the *P. graminis* f. sp. *tritici* effector AvrSr35 with a mature length of 578 amino acids (Salcedo *et al*., 2017).

With these issues in mind, bioinformatic pipelines have been developed to encompass multiple criteria to refine effector prediction.

### 3.6 Bespoke bioinformatic pipelines

Saunders *et al*. developed an in silico analysis pipeline that moved away from reliance on sequence similarity‐based methods for effector identification and included physiological functions such as expression profiles, taxonomic information, and genomic features of potential candidates (Saunders *et al*., 2012b). To identify the repertoire of potential effectors within two rust fungus genomes, a clustering algorithm grouped candidates into families and ranked their likelihood of being effectors based on the knowledge that filamentous pathogen effectors have a least one of eight specific properties. These properties included the absence of recognized Pfam domains, similarities to haustorial proteins, and the presence of internal repeats. The number of candidates continued to functional analysis using this pipeline was greatly reduced (Saunders *et al*., 2012b). This approach has limitations as it is dependent on the thresholds based on a priori assumptions about effector properties; the number of missed effectors remains to be seen.

At each step of the general pipeline for effector prediction and subsequent characterization, in silico tools, whether bioinformatical software or web‐based servers, have been developed to aid effector refinement. The presence of signal peptides, transmembrane motifs, or GPI anchors can all be predicted using tools such as SignalP (www.cbs.dtu.dk/services/SignalP/), TMHMM (www.cbs.dtu.dk/services/TMHMM/), and PredGPI (gpcr.biocomp.unibo.it/predgpi/pred.htm), which use neural networks or hidden Markov modelling to recognize motifs within protein sequences associated with these features (Pierleoni *et al*., 2008; Armenteros *et al*., 2019). The subcellular localization of candidate effectors can also be predicted by searching for chloroplast or mitochondrial transit peptides or nuclear localization signals using tools such as WoLF‐PSORT (wolfpsort.hgc.jp/) or LOCALIZER (localizer.csiro.au/) (Horton *et al*., 2007; Sperschneider *et al*., 2017). Machine learning has also resulted in the development of web‐based tools that can predict with 89% accuracy whether proteins in the predicted secretome are effectors or not. EffectorP2.0 (effectorp.csiro.au/) takes into account the net charge and serine/cysteine content of proteins to prioritize candidate effectors for further functional validation (Sperschneider *et al*., 2018).

### 3.7 Genomic landscape and transposable elements

Many fungal plant pathogens exhibit a two‐speed genome, with distinct genomic compartments evolving at different rates. Alongside core stable regions, which are slow to evolve and often contain genes involved in metabolism, are hypervariable areas with high recombination and richness in repetitive sequences, including transposable elements (TEs). This genomic landscape and the presence of TEs serve to drive adaptive evolution (Faino *et al*., 2016) and these hypervariable regions often are the location of genes associated with pathogenicity, including effectors (Fouché *et al*., 2018; Jones *et al*., 2018).

In *M. oryzae* and *Zymoseptoria tritici,* TEs are associated with pathogenicity clusters and are seen to flank the first characterized *Z. tritici* effector, AvrStb6 (Bao *et al*., 2017; Zhong *et al*., 2017). TEs have also been shown to interfere with effector gene expression via epigenetic control. For example, AvrLm1 in *Leptosphaeria maculans*, located in a TE‐rich genomic region, showed distinct histone methylation that acts to temporarily suppress expression during colonization to evade host recognition (Soyer *et al*., 2014; Fouché *et al*., 2018). This suggests that the variability of the genomic region or the proximity to TEs maybe useful factors in refining the search for candidate effectors.

Following the sequencing, genome assembly and annotation of the tumour‐forming maize smut fungus *Ustilago maydis*, c.18% of genes encoding secreted proteins were found to be arranged into 12 discrete clusters within the genome (Kämper *et al*., 2006). These clusters were co‐regulated by a central pathogen‐development regulator and expression induced in tumour tissue. Deletions of five clusters caused clear changes in virulence, including the largest cluster, 19A, which caused a strong attenuation in virulence and reduced tumour formation upon deletion (Kämper *et al*., 2006; Brefort *et al*., 2014). Subsequent subdeletions of 19A members led to the identification of the effector Tin2, required for anthocyanin production (Brefort *et al*., 2014; Tanaka *et al*., 2014).

### 3.8 Comparative genomics

By comparing the genomes of *U. maydis* and *Sporisorium reilianum*, Schirawski *et al*. (2010) found that effector clusters and pathogenicity‐related regions were more highly diverged between the close relatives than the rest of the genome. This comparison led to the identification of the *pit* gene cluster involved in tumour formation in *U. maydis* (Doehlemann *et al*., 2011). Within this cluster the secreted effector Pit2, involved in plant defence suppression and cysteine protease inhibition, was found (Doehlemann *et al*., 2011; Mueller *et al*., 2013). This same comparison was used to locate gene clusters and candidate effectors in *S. reilianum*, and whilst genes that have a partial impact on disease severity have been identified, as yet no candidates strongly attenuate virulence (Ghareeb *et al*., 2019).

### 3.9 Lineage‐specific elements

Novel effectors were identified in the asexual fungus *V. dahliae*, where chromosome reshuffling has led to the formation of lineage‐specific (LS) regions of plasticity in the genome (de Jonge *et al*., 2013). These LS regions are enriched with retrotransposon and repetitive sequence elements, as well as being the location of many candidate effectors. Contrary to the two‐speed genome hypothesis, these LS regions show strong levels of conservation with little to no single nucleotide polymorphisms (SNPs) being identified, even within the intergenic regions (Depotter *et al*., 2019). In one such LS region, four putative effectors were identified, including the LysM domain containing effector Vd2LysM, which was only found in the VdLs17 strain (de Jonge *et al*., 2013).

### 3.10 Sequence divergence

Molecular variation in filamentous phytopathogen genes is known to be essential for altering pathogen–host interaction outcome and can provide insight into the evolution of virulence (Allen *et al*., 2008). Polymorphisms in effector sequences among isolates can impact on virulence and are involved in host adaptation; this makes them promising targets for disease control strategies.

The genomes of four isolates of the wheat yellow stripe rust fungus *Puccinia striiformis* f. sp. *tritici* were resequenced and assessed for SNPs. Proteins that displayed nonsynonymous substitutions between isolates that differed in virulence on specific wheat cultivars were identified (Cantu *et al*., 2013). This led to five secreted polymorphic candidate effectors being nominated for further characterization from a predicted secretome of 2,999 proteins.

This sequence divergence has also proved useful in identifying pathogens in the field. Using the Oxford Nanopore MinION sequencer, 242 highly variable genes were used to collect real‐time population dynamics data of *P. st* *riiformis* f. sp. *tritici* isolates in Ethiopia (Radhakrishnan *et al*., 2019). This Mobile And Real‐time PLant disEase (MARPLE) diagnostic system can be used to monitor the emergence of plant pathogen strains, but can also be adapted to include newly characterized effectors within the panel of genes. Going forward, MARPLE will allow for the monitoring of mutations and the detection of effector evolution that may be linked to gain of virulence of phytopathogens, all within the confines of the field.

### 3.11 Association mapping in the sequencing era

In silico predictions of effectors, whilst allowing us to rapidly screen whole genomes for candidates, lack discriminatory power and often result in candidate effectors having no clear impact on pathogen virulence. Genome‐wide association studies (GWAS) and quantitative trait locus (QTL) mapping can identify loci associated with heritable phenotypic variation, such as virulence, thereby complementing techniques to identify and clone Avr effectors recognized by known host resistance proteins (Plissonneau *et al*., 2017). The *Zymoseptoria tritici* effector AvrStb6 was isolated in this way (Zhong *et al*., 2017). Using crosses between two Swiss strains of *Z. tritici,* QTL mapping found a confidence interval containing nine candidates for *AvrStb6*. Combining this with a GWAS study from over 100 different natural isolates led to one candidate, a small cysteine‐rich secreted protein that was not present in the original *Z. tritici* genome annotation (Zhong *et al*., 2017).

An additional benefit of using GWAS in effector discovery is that the natural variation in SNP calling identified in wild populations can be used to quantify how each SNP contributes to pathogen virulence (Sánchez‐Vallet *et al*., 2018b). Integrating GWAS with transcriptome dataset, referred to as transcriptome‐wide association studies (TWAS) (Wainberg *et al*., 2019), identified the link between genes and traits across populations and has been used to discover *Blumeria graminis* f. sp. *hordei* Avr_a_ effectors, including Avr_a9_ (Saur *et al*., 2019a).

## 4 FUNCTIONAL CHARACTERIZATION

Make your work to be in keeping with your purpose.

Leonardo da Vinci, *The Practice of Painting*

### 4.1 Knock out or knock down: let's be disruptive

One of the simplest ways to determine the pathogenicity of a candidate effector is to disrupt the encoding gene and determine whether the virulence on a susceptible host or the Avr phenotype on a resistance genotype is compromised. Early transformation studies of the *C. fulvum* effectors relied on double homologous recombination to insert a selectable marker into the target gene encoding a known effector such as *ecp1* and *ecp2*, thus disrupting them (Laugé *et al*., 1997). Later sequencing technology allowed transformations without the need for cloning. Mutants of the corn smut fungus *Ustilago maydis* were made using PCR‐based protocols combined with protoplast transformation to generate candidate effector knockout mutants (Schulz *et al*., 1990; Kämper, 2004). This method is widely used and has successfully facilitated the functional characterization of *U. maydis* effectors, including Rsp3 and Cce1 (Ma *et al*., 2018a; Seitner *et al*., 2018).

*Agrobacterium tumefaciens*‐mediated transformation (ATMT) is another method to disrupt genes and is widely used in plant transformations. ATMT was first used in fungi in budding yeast in 1995 and then the technique was adapted for use in filamentous fungi, including *M. oryzae* (Bundock *et al*., 1995; Rho *et al*., 2001). This method relies on the targeted insertion of a selectable marker into the fungal genome from a disarmed Ti plasmid of transformed *Agrobacterium* to disrupt the gene of interest. The selectable marker is incorporated into the fungal genome via homologous recombination, a process that occurs easily in yeast. This mechanism, however, is highly variable in filamentous fungi, where nonhomologous end‐joining (NHEJ) appears to be the dominant DNA repair pathway over homologous recombination (Meyer *et al*., 2007; Villalba *et al*., 2008). The Ku70 protein is part of a complex that regulates the NHEJ pathway (Ninomiya *et al*., 2004), and its deletion has led to the increase of homologous recombination in *M. oryzae* from <25% to 80% (Kershaw and Talbot, 2009). Combining ATMT with the generation of ∆*Ku70* mutants led to the characterization of the *Z. tritici* Avr effector AvrStb6 (Zhong *et al*., 2017).

Another, more recent, method of gene disruption is using the genome‐editing system CRISPR‐Cas9. Originally identified as an immune mechanism in bacteria and archaea, the CRISPR‐Cas9 system is used as a genome‐editing tool in plants and animals, and was adapted by Nødvig *et al*. (2015) for use in filamentous fungi (Mali *et al*., 2013; Fauser *et al*., 2014; Nødvig *et al*., 2015). This technique has led to targeted gene disruption and consequent characterization of effectors in the oomycete *P. sojae* and the fungal pathogen *U. maydis* (Fang and Tyler, 2016; Schuster *et al*., 2018).

**FIGURE 2:**
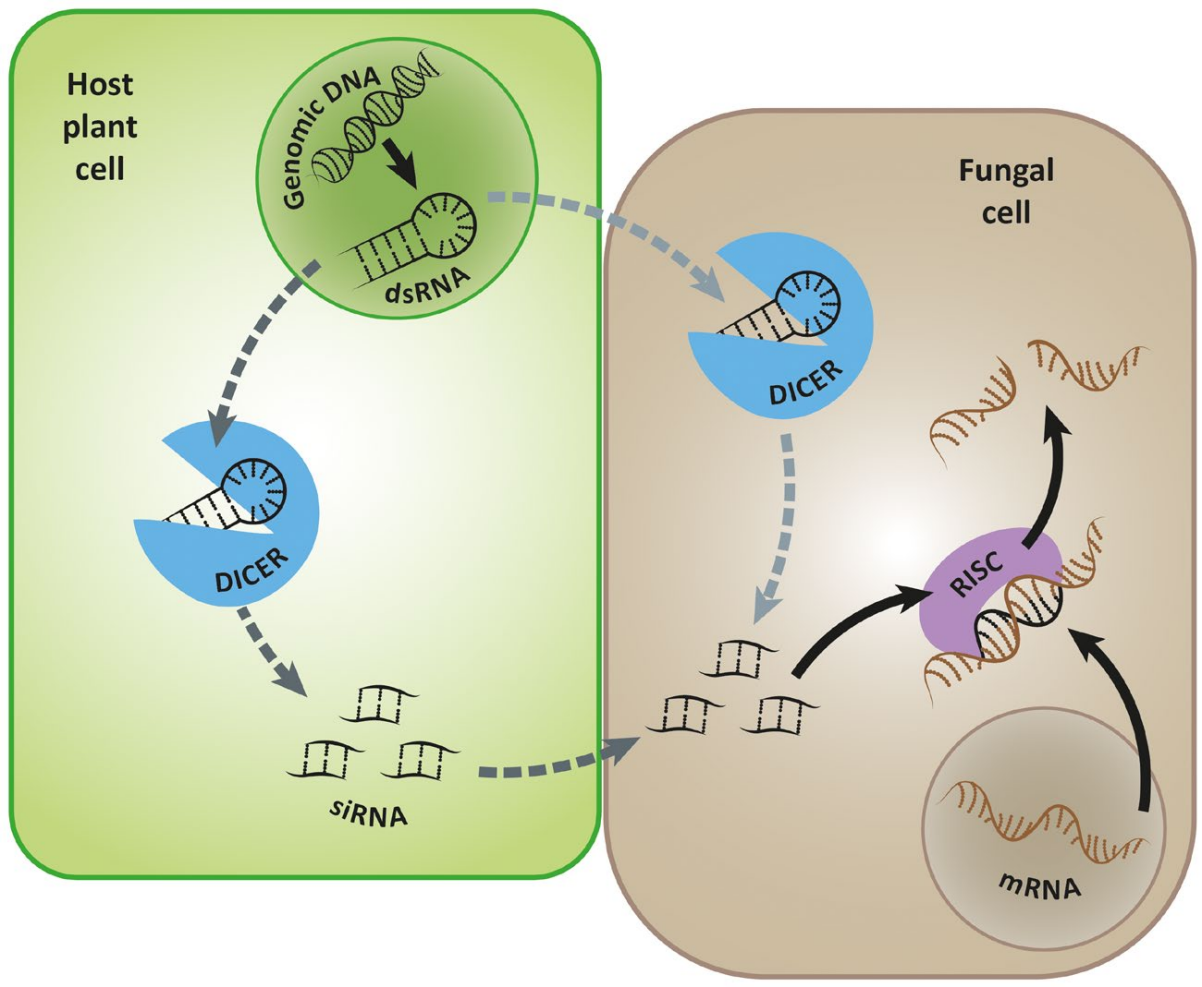
The host‐induced gene silencing (HIGS) construct encodes an inverted sequence that forms a hairpin double‐stranded (ds) RNA following transcription and is introduced into the host plant either by transient or stable transformation. The dsRNA is processed to form small interfering RNA (siRNA), either before or after delivery to the pathogen cell using the plants innate RNAi machinery. Once inside the fungal cells the siRNA silences the target effector genes by interfering with the target mRNA transcripts (Koch *et al*., 2018). The movement of small RNA between host and pathogen is detailed by Wang and Dean (2020).

There are, however, difficulties in producing stable transformants in phytopathogens that are obligate biotrophs (Thomas *et al*., 2001; Lorrain *et al*., 2019). In these cases, knockdown technologies such as host‐induced gene silencing (HIGS) are more successful. The HIGS assay detailed in Figure 2 has led to the identification of many effectors, including the barley powdery mildew *Blumeria graminis* f. sp. *hordei* ribonuclease‐like effectors BEC1054 and BEC1011 (Nowara *et al*., 2010; Pliego *et al*., 2013; Pennington *et al*., 2019).

Gene disruption assays do have their limitations even when successful transformants are produced. Many effector mutants display no associated phenotype. Genetic redundancies, where multiple effectors have the same function, or buffering, where the host compensates or interfers in signalling using alternative pathways, may result in false‐negative results (Hillmer *et al*., 2017; Tyler, 2017).

### 4.2 In planta expression

When a candidate effector is heterologously expressed in planta various functional assays can be used to determine the virulence activities of the protein.

Necrosis assays monitor for the induction of HR‐like cell death, which can be a result of Avr/R protein/guardee protein interactions or be directly induced by the candidate effector. These assays were first carried out using the model plant *Nicotiana tabacum* (tobacco), which is infiltrated with transformed *Agrobacterium* that delivers the effector gene expressed from an inducible promoter into the plant cell for transient protein production (Kamoun *et al*., 1999; Qutob *et al*., 2002; Ma *et al*., 2012).

In 1999 the *P. infestans* and *C. fulvum* effectors *Inf1* and *Avr9*, respectively, were transformed into either wild‐type or *Cf‐9* transgenic *N. tabacum* using this method. The assay showed that INF1 was capable of inducing necrosis in wild‐type tobacco whilst Avr9 could only do so in transgenic tobacco expressing the corresponding R gene *Cf‐9* (Kamoun *et al*., 1999). Later *Avr9* and *Cf‐9* were transiently coexpressed in *N. tabacum* using agroinfiltration to confirm the induction of HR in the nonhost plant following expression of the Avr/R gene pairs (Van der Hoorn *et al*., 2000).

Effector characterization in nonhost dicotyledonous model plants maybe more suited to high‐throughput screening than in cereal hosts. However, these highly artificial scenarios do have several limitations. A negative screen with no visible phenotype upon recombinant expression may indicate either the candidate is not an effector or the effector target/receptor is lacking in the model species. On the otherhand, HR‐induced necrosis in an effector screen may not be caused by a specific effector/target interaction but by nonhost resistance (NHR) triggered by detection of the candidate (Kettles *et al*., 2017). Although of interest, by definition the latter scenario would not occur in native host interactions. Therefore, expression assays in the native host maybe the more useful for functional characterization.

Candidate effectors can be transiently expressed in protoplast cells and cell death monitored via the reduction in expression of a co‐transfected reporter gene such as β‐glucuronidase (GUS) or luciferase (Chen *et al*., 2006; Lu *et al*., 2016). This approach was used to identify the cell death‐inducing properties of five *M. oryzae* effectors, including MoCDIP4 (*M. oryzae* cell death inducing protein 4), in rice protoplasts (Chen *et al*., 2012) and the NLR‐mediated recognition of four newly identified barley powdery mildew avirulence effectors, including AVR_a9_, in barley (Saur *et al*., 2019a).

Cell‐death suppression assays are used to detect the alteration of the plant immune response induced by a known cell death elicitor. The overexpression of the stem rust candidate effector PSTha5a23 in *Nicotiana benthamiana* suppresses *P. infestans* INF1‐triggered cell death, indicating that PSTha5a23 plays a role in controlling plant defence responses (Cheng *et al*., 2017).

An alternative method of expressing effectors in plant cells uses the bacterial type III secretion system (T3SS) derived from the tomato bacterial speck pathogen *Pseudomonas syringe* pv. *tomato* DC3000 (He *et al*., 2004). This system was first adapted for filamentous plant pathogens by Sohn *et al*. (2007) to deliver oomycete effector proteins into *Arabidopsis*. Sohn *et al*. showed that, by fusing the downy mildew (*H. parasitica*) effectors ATR1 and ATR13 to the N‐terminal secretion‐translocation signals of the *P. syringae* effectors AvrRpm1 and AvrRps4, the effectors could be secreted into *Arabidopsis* plant cells and contribute to pathogen virulence. Since then, the T3SS has been used to functionally characterize candidate effectors from multiple oomycetes, including *P. infestans* and *Hyaloperonospora arabidopsidis* (Whisson *et al*., 2007; Fabro *et al*., 2011). Despite T3SS being used to screen effector candidates of stem rust (*P. graminis* f. sp. *tritici)* and bean rust (*Uromyces appendiculatus*), this system is rarely used for fungal effector characterization and has limited success on cereals (Upadhyaya *et al*., 2014; Qi *et al*., 2019; Saur *et al*., 2019b). These problems are linked to the required unfolding and refolding of effectors prior to insertion, especially those rich in cysteine–cysteine bridges.

As well as monitoring for necrosis, or lack thereof, the in planta growth of another pathogenic species can be used as a proxy to determine the role in virulence effectors play. Stable transformants of the nonhost *Arabidopsis* that expressed candidate poplar rust fungus (*M. larici‐populina*) effectors were inoculated with the oomycete pathogen *H. arabidopsidis*. Eleven of 16 effectors tested supported greater sporulation of this native *Arabidopsis* pathogen, suggesting that the effectors had the capacity to interfere with processes in a nonhost plant to favour pathogenesis (Germain *et al*., 2018).

### 4.3 The viral overexpression system

Due to the limited effectiveness of both T3SS and *Agrobacterium*‐mediated transient expression in most cereal species, viruses have been developed as efficient vectors for heterologous protein expression (viral overexpression, VOX) (Lee *et al*., 2012).

**FIGURE 3:**
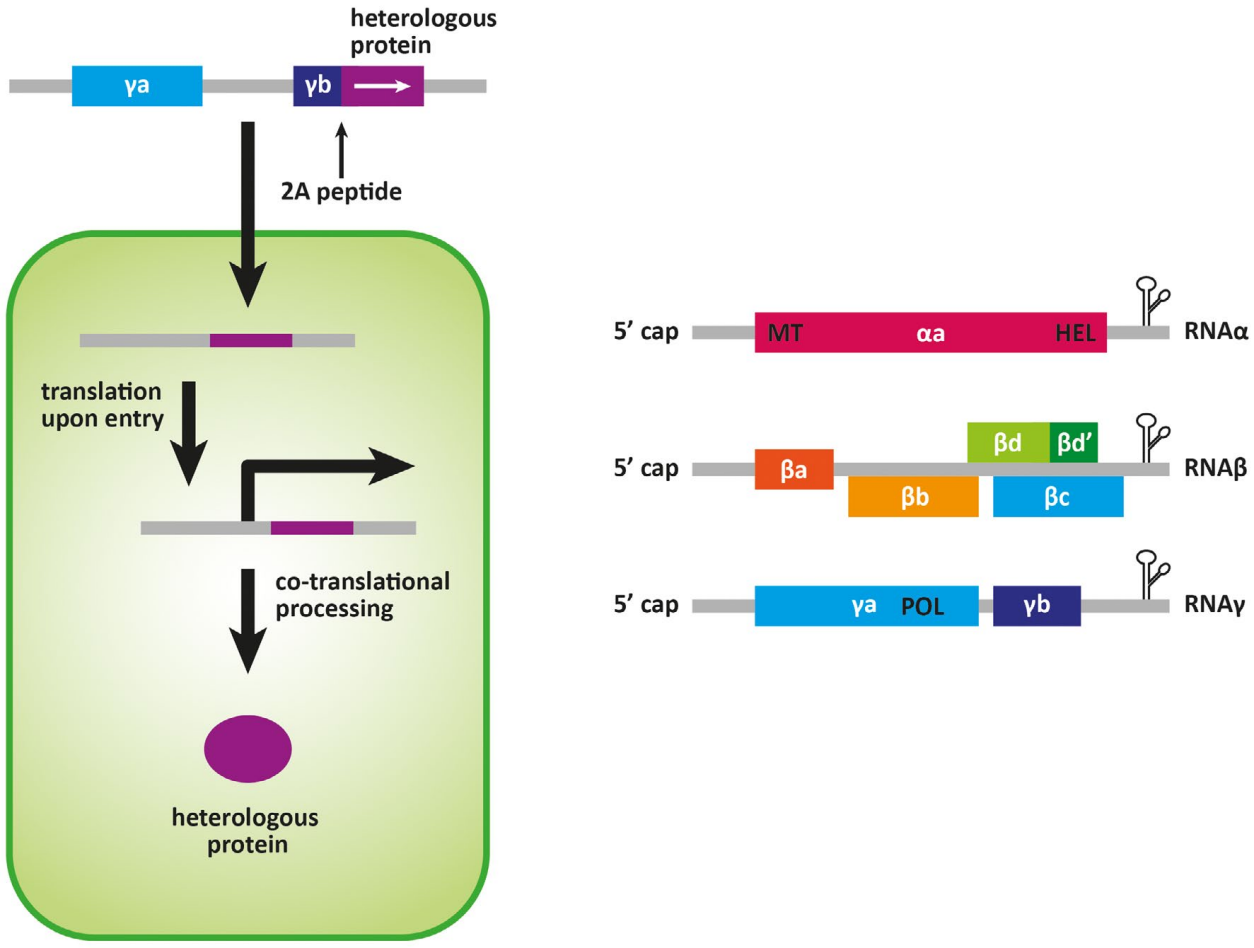
The BSMV‐VOX technology adapted from Lee *et al*. (2012). (a) Virus‐mediated overexpression (VOX) system. The heterologous protein coding sequence is inserted in the γ genome of barley stripe mosaic virus (BSMV), upstream of the in‐frame stop codon in the *γb* open reading frame (ORF). A gene for the autoproteolytic peptide 2A is also inserted between the 3′ terminus of the *γb* ORF and the gene of interest for processing the fusion protein during translation, thus releasing the heterologous protein of interest. (b) The BSMV genome is composed of three RNAs that are capped at the 5′ end and form a tRNA‐like hairpin secondary structure at the 3′ terminus. RNAα encodes the αa replicase protein containing methyltransferase and helicase domains. RNAβ encodes coat and movement proteins whilst RNAγ encodes the polymerase (POL) component of replicase, and the cysteine‐rich γb protein involved in viral pathogenicity.

The barley stripe mosaic virus (BSMV) was first verified as a tool for protein expression when used to overexpress the luciferase reporter gene in protoplast cells and later to express green fluorescent protein (GFP) in planta (Joshi *et al*., 1990; Haupt *et al*., 2001; Lawrence and Jackson, 2001). The BSMV vector was adapted for use in the VOX system and used to characterize the function of the fungal effector ToxA (Manning *et al*., 2010) (Figure 3). However, the compact nature of the virus results in a negative correlation between fragment size and stability of the viral vector (Avesani *et al*., 2007; Bruun‐Rasmussen *et al*., 2007). BSMV‐VOX has been widely used for heterologous expression of proteins up to 150 amino acids; however, as previously stated there is no agreed size limit for an effector (Figure 3a; Bouton *et al*., 2018).

Another limitation of BSMV for use in effector discovery is that this virus has a tripartite RNA genome (Figure 3b). The heterologous protein is inserted into the γ genome yet all three subgenomes are required to combine for successful expression in planta making BSMV‐VOX unsuitable for high‐throughput screening assays.

The foxtail mosaic virus (FoMV) has been adapted for use in VOX systems in cereals (Bouton *et al*., 2018). Vectors derived from FoMV such as PV101 avoid many of the caveats of those from BSMV. FoMV has a monopartite RNA genome and the PV101 vector can be used to successfully express proteins up to 600 amino acids in size. In addition, unlike BSMV vectors, PV101 allows for heterologous expression of proteins in their native form, including possible signal peptides, without the need for processing from proteases that may only be 90% efficient (Bouton *et al*., 2018). In situations where the effector expressed from the VOX vector rapidly triggers R protein‐mediated defences, virus spread is halted and therefore the phenotypic readout in the bioassay is the lack of systemic spread of the recombinant virus (Saintenac *et al*., 2018).

### 4.4 Where do they go?

Knowing the localization of candidate effectors within host tissues not only demonstrates that the protein can be translocated from the pathogen to its host, but also suggests where the effector target(s) may be found. Traditionally in situ hybridization assays were done where antibodies were raised against the effector or an added epitope tag and detected using transmission electron microscopy (TEM). Translocation of fungal effectors into the host cell was first shown using an immunocytochemical approach in rusts. The gold‐ and fluorescence‐labelling of four independently raised antibodies to the RTP1p protein in *Uromyces fabae* and its homolog in *Uromyces striatus* showed that in later stages of infection RTP1p translocated from the extrahaustorial matrix to inside the plant cell itself (Kemen *et al*., 2005).

For apoplastic effectors, localization was often determined by means of their isolation. The *C. fulvum* effectors Avr2, Avr4, Avr9, and Ecp6 were directly isolated from the apoplastic fluid, whereas the *P. infestans* protease inhibitor EPIC1 was isolated from the apoplast after antibodies were raised (Joosten *et al*., 1997; Rooney *et al*., 2005; Tian *et al*., 2007; Bolton *et al*., 2008). Whilst successful, these approaches are laborious, expensive, and not suited to high‐throughput screening of either apoplastic or cytoplasmic effector candidates (Dalio *et al*., 2017).

The nuclear localization of the *P. infestans* CRN effectors was determined using N‐terminal GFP tagging and confocal microscopy. By overexpression five GFP‐CRN (without the signal peptide) fusion proteins in planta the effectors were shown to accumulate within plant cell nuclei (Schornack *et al*., 2010). High‐throughput screening of 61 candidate effectors (ChECs) from the anthracnose fungus *Colletotrichum higginsianum* using this method found that whilst nine of the ChECs were imported into the nucleus, others localized to the Golgi bodies, microtubules, and peroxisomes, all novel targets for fungal effectors (Robin *et al*., 2018).

The *U. maydis* effectors Cmu1 and Tin2 have been shown to localize to the maize cytoplasm; however, this could not be demonstrated when fluorescently tagged (Djamei *et al*., 2011; Tanaka *et al*., 2014; Tanaka *et al*., 2015). This may be due to the tags inhibiting the partial unfolding of the effectors, thereby preventing their translocation, or the incorrect refolding of the tags themselves upon entering the cytoplasm (Lo Presti *et al*., 2015).

Whilst investigating the translocation of *M. oryzae* effectors into rice cells, fluorescent‐tagged cytoplasmic effectors were seen to first accumulate in the plant‐membrane derived infection structure, the biotrophic interfacial complex (BIC), prior to delivery into the cytoplasm, whereas tagged apoplastic effectors localized to the invasion hyphae (Mosquera *et al*., 2009; Khang *et al*., 2010). The BIC’s role in effector translocation could only be confirmed by the addition of a nuclear localization signal (NLS) to cytoplasmic effectors, causing artificial accumulation in the nucleus of the neighbouring rice cells. This approach concentrated the fluorescent signal into discrete foci observable using live cell imaging (Khang *et al*., 2010).

For apoplastic effectors it is difficult to distinguish between apoplastic or cytoplasmic localization when the fluorescently tagged candidates appear to localize to the plasma membrane or cell wall. Enlarging the apoplastic space by the stepwise addition of hypertonic solutions, a process known as plasmolysis, revealed that the *U. maydis* host‐peroxidase inhibitor Pep1 was indeed apoplastic and was evenly distributed throughout the enlarged space (Oparka, 1994; Doehlemann *et al*., 2009).

Alternatively, the BirA assay does not require the use of large fluorescent tags that may interfere with effector function or localization. BirA, developed by Lo Presti *et al*., is based on the bacterial enzyme biotin ligase that biotinylates any protein that has a short (15 amino acids) peptide Avitag (Lo Presti *et al*., 2017). Maize lines that expressed the biotin ligase in the cytoplasm were infected with transformed *U. maydis* strains that had either the Cmu1 or the Tin2 effectors tagged with the Avitag. Biotinylation was detected via immunoprecipitation of extracted proteins using streptavidin‐coated magnetic beads, thus confirming the tagged effectors had met the biotin ligase in the host cytoplasm (Lo Presti *et al*., 2017).

## 5 EFFECTOR INTERACTIONS

…to manage a system effectively, you might focus on the interactions of the parts rather than their behavior taken separately.

Russell L. Ackoff and Fred Emery, *On purposeful systems*

Arguably the Holy Grail of effector characterization is to identify the exact molecular targets of each effector and/or the molecules used by the plant to bind to them. This can lead to defining the precise sequences and molecular interactions occurring at the point(s) of direct contact. The former is very challenging because the effector sequences do not give many clues as to their function(s).

### 5.1 A shot in the dark: unbiased screening

Unbiased “forward” screening to find protein–protein interactions (PPI) is a common technique used in many aspects of molecular biology. The yeast two‐hybrid system (Y2H), first developed 30 years ago, allows for the large‐scale screening of cDNA libraries derived from pathogen‐infected plants for effector target identification (Fields and Song, 1989; Mukhtar *et al*., 2011). Interactions detected by Y2H screens must be validated by additional PPI assays as this approach is prone to false positives.

**FIGURE 4:**
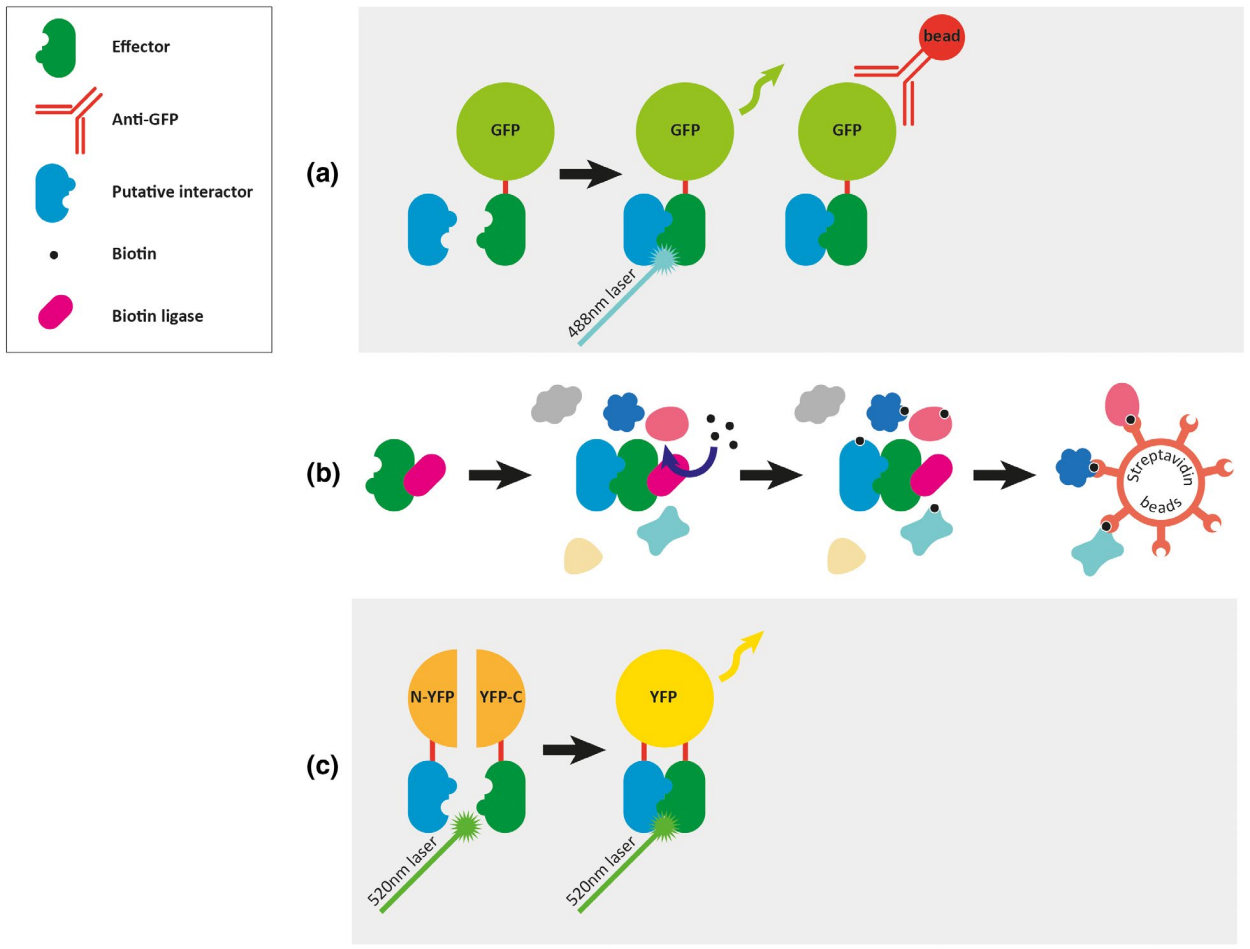
Protein–protein interaction techniques. (a) Co‐immunoprecipitation, effectors are tagged with a peptide sequence such as green fluorescent protein (GFP) and expressed in planta. Antibodies are used to pull down the protein complexes that can then be analysed using liquid chromatography and mass spectrometry (LC‐MS/MS) (Petre *et al*., 2017). (b) Biotinylation, effectors are fused to mutant biotin ligase enzymes and expressed in vivo. The fusion protein catalyses the biotinylation of interacting and proximal proteins in the presence of biotin. The biotinylated proteins are captured using streptavidin beads (Roux *et al*., 2012). (c) Bimolecular fluorescence complementation, the effector and putative interactors are tagged with nonfluorescent fragments of yellow fluorescent protein (YFP). Direct interaction of the tagged effectors results in YFP reassembly visualized in vivo or quantified using flow cytometry (Kerppola, 2008; Graciet and Wellmer, 2010; Miller *et al*., 2015).

The most common Y2H validation technique is co‐immunoprecipitation (Co‐IP). Co‐immunoprecipitation is used to screen effector interactors in heterologous systems. When 20 candidate poplar rust fungus (*M. larici‐populina*) effectors were tagged with GFP and expressed in *N. benthamiana,* five were found to specifically interact with plant proteins by pull‐down assays using anti‐GFP followed by protein purification (Figure 4a) (Petre *et al*., 2015).

Biotinylation is also used for proximity labelling based on tools such as BioID (Li *et al*., 2017). A benefit of proximity labelling over co‐immunoprecipitation is the possibility of identifying proteins that only weakly or transiently interact with the target (Figure 4b). Recently a new proximity labelling tool, TurboID, has been shown to provide more efficient labelling in planta compared to BioID and can also reduce the biotin incubation time from 16 hr to 10 min (Branon *et al*., 2018; Zhang *et al*., 2019). These new advances in PPI technology pave the way for higher‐throughput effector interaction screening in planta.

### 5.2 Split‐marker complementation

The effector Pep1 is essential for the pathogenicity of the corn smut fungus *U. maydis* (Doehlemann *et al*., 2009). The direct interaction between Pep1 and the plant peroxidase POX12 was validated using the bimolecular fluorescence complementation (BiFC) assay (Figure 4c), which involves two parts of a fluorescent marker being fused to candidate interactors. Only when the interactors meet can the full‐length fluorescent marker assemble and be detected. Alternatively, the firefly‐derived enzyme luciferase can be used for split‐marker complementation. This has the advantage over BiFC for in planta studies because luciferase does not require excitation by light for detection, thereby eliminating autofluorescence interference (Li *et al*., 2011). However, using split‐marker complementation for PPI validation is not infallible as heterologous overexpression of proteins in *N. benthamiana* can affect protein localization and therefore interactors.

### 5.3 Structural interactions: pinpointing the surface contacts and their strengths

Knowledge of effector structures whilst in complex with their targets gives us a greater insight into the molecular basis of these cross‐kingdom interactions.

The *C. fulvum* effector Avr4 was one of the first to be characterized from a family of effectors that bind to and protect fungal cell‐wall chitin from host chitinase (Joosten *et al*., 1997; van den Burg *et al*., 2006). Recently the crystalline structure of Avr4 in complex with its chitin ligand (resolved to 1.95Å) has highlighted the residues required for this function (Hurlburt *et al*., 2018). Structural mutant studies have also shown that recognition of the Avr4 by the cognate Cf‐4 immune receptor does not depend on the same ligand binding as previously thought (Hurlburt *et al*., 2018).

The crystal structure of the rice intracellular NLR immune receptor Pik in complex with the *M. oryzae* effector Avr‐Pik (1.6Å resolution) reveals molecular details of the recognition event that leads to HR‐induced cell death (Maqbool *et al*., 2015). The effector surface involved in this interaction was also identified as being involved in the surface interactions between Avr‐Pia and the NLR‐RATX1 in *M. oryzae* (Ortiz *et al*., 2017).

In the past decade protein structures are increasingly being resolved without the need to form crystals or use damaging X‐rays but by using cryo‐electron microscopy. This technique is widely used to resolved proteins in complexes and has been used to show both inactive *Arabidopsis* NLR complex ZAR1‐RKS1 and the intermediate form when the complex interacts with a protein modified by the bacterial effector AvrAC (*Xanthomonas campestris* pv. *campestris*) (Wang *et al*., 2019). Cryo‐e, despite gaining popularity in structural biology, is unable to resolve proteins smaller than 65 kDa, a size exclusion that would include many fungal and oomycete effectors (Muench *et al*., 2019).

The strength of effector–target interactions can be determined using isothermal titration calorimetry whereby direct measurement of the heat that is either released or absorbed during the molecular binding event gives a complete thermodynamic picture of the reaction, including affinity, enthalpy, and stoichiometry (Duff *et al*., 2011). For the conserved *M. oryzae* MAX effector Avr1‐CO39, isothermal titration calorimetry was used to confirm that direct interaction with the heavy‐metal associated (HMA) domain of the rice NLR RGA5 was required for effector binding (Guo *et al*., 2018).

A greater understanding of how structural interactions aid the specificity of Avr recognition is vital for future work in developing sustainable disease resistance in important food crops.

## 6 EXPLOITING EFFECTOR DISCOVERIES TO CONTROL CROP PLANT DISEASES

Knowing is not enough; we must apply. Willing is not enough; we must do.

Johann Wolfgang von Goethe, *Wilhelm Meister's Journeyman Years*

The ultimate goal of effector discovery, from identification to characterization to target interactions, is to apply this knowledge to the control of multiple pathogens that threaten our food security.

### 6.1 “Effectoromics”

For over 100 years disease resistance loci have been introduced into crops and subsequently shuffled through traditional breeding techniques, whether that be as individual genes or stacked to achieve often only short‐lived resistance to pathogens (Vleeshouwers *et al*., 2011; Langner *et al*., 2018). Despite this, the search for novel *R* genes with durable or broad‐spectrum resistance remains ongoing.

The term “effectoromics” is used to describe the use of effectors in high‐throughput screening for R protein function in either the germplasm of crop cultivars or a sexually compatible species. Avr effectors can be harnessed to screen rapidly for HR phenotypes, a hallmark of an ETI response (Vleeshouwers and Oliver, 2014). Well‐established techniques of transient overexpression of Avrs using viral vectors such as potato virus X (PVX) in conjunction with agroinfiltration have been widely used for the identification and cloning of *R* genes in solanaceous species such as potato, tomato, and wild *Solanum* species (Takken *et al*., 2000; Du *et al*., 2014).

The search for broad‐spectrum or more robust *R* genes for breeding purposes maybe more nuanced than previously thought as multiple unrelated *R* genes can recognize the same pathogen effector (Aguilera‐Galvez *et al*., 2018).

### 6.2 Screening with necrosis‐inducing effectors to remove host susceptibility loci

The necrosis‐inducing effector ToxA was isolated from the wheat tan spot fungus *P. tritici‐repentis* in 1996. Infiltration of purified ToxA into the apoplastic space of a susceptible wheat cultivar containing the *Tsn1* susceptibility (*S*) gene is itself sufficient to induce tan spot symptoms (Tomas *et al*., 1990; Ballance *et al*., 1996; Ciuffetti *et al*., 1997; Welti and Wang, 2004). Wheat breeders routinely use the purified toxin to screen all new wheat germplasm to eliminate susceptible lines from their breeding programmes. This method is preferred over screening for molecular markers linked to the corresponding *Tsn1* locus due to the ease of application and speed of results (Vleeshouwers and Oliver, 2014). *Tsn1* removal from all newly commercially released wheat varieties has improved resistance to tan spot disease and Australia has seen a 26% reduction in ToxA‐sensitive wheat grown in the 10 years prior to 2016 (See *et al*., 2018).

## 7 KEEPING TRACK OF EFFECTOR DISCOVERIES IN MULTIPLE SPECIES IN AN INCREASINGLY DATA‐RICH WORLD

A place for everything, and everything in its place.

Idiom from 17th century

In the past two decades effector discovery and characterization have exploded with regard to crop pests and pathogens. This key information is found in multiple original research publications, review articles, UniProt, individual pathogen genome browsers, and species‐specific websites. However, to aid future research and guide the direction of work the genotype and fine phenotyping data surrounding these discoveries and new insights needs to be FAIR (Findable, Accessible, Interoperable, and Reusable) to molecular plant pathologists as well as the wider life sciences communities.

Publicly available repositories of curated data regarding proteins with confirmed roles in pathogenicity and virulence are a fundamental tool for effector study. The Pathogen–Host Interactions database (PHI‐base, www.phi‐base.org) is a manually curated database comprising over 6,780 genes from 268 pathogens of over 210 hosts (September 2019), of which 60% are plants (Urban *et al*., 2020). Within the PHI‐base (version 4.8), 799 interaction entries involve 731 distinct functionally characterized fungal or oomycete effectors from over 40 species. Collectively, these effector entries and their considerable metadata can be used for comparative studies, genome landscape explorations, the enrichment of transcriptome/proteome data sets, PPI network predictions, as well as the starting point for potentially novel artificial intelligence approaches.

## 8 CONCLUSIONS AND OUTLOOK

“Would you tell me, please, which way I ought to go from here?”

“That depends a good deal on where you want to get to,” said the Cat.

Lewis Carroll, *Alice in Wonderland*

Effectors are the mysterious molecular tools evolved and used by plant pathogens in multiple ways. Effector studies are of vital importance in addressing the global food security challenge, yet the explosion in research efforts aimed at understanding effector biology over the last few decades has left us with a dichotomy in our knowledge. Due to early focus on a small number of pathosystems, whether due to experimental convenience or the economic impact of the disease, for some pathogens, such as *M. oryzae,* we have resolved three‐dimensional protein structures and know interacting surfaces of multiple effectors and their interactors. In other cases, important crop pathogens such as *F. graminearum* and the newly emerging pathogens *Ramularia collo‐cygni* and *Corynespora cassiicola*, although several hundred candidate effectors have been predicted, each lacks functional characterization (McGrann *et al*., 2016; Lopez *et al*., 2018).

The arrival of full genome sequencing almost two decades ago has been a double‐edged sword. Bioinformatic pipelines and the development of prediction software has sped up the refinement of putative effectors whilst simultaneously highlighting the vastness of the gene repertoires to be investigated. For effector characterization, the future efficiency not only depends on the development of ultrahigh‐throughput functional assays but also their use in combination with lower‐throughput novel and well‐established techniques such as QTL mapping and GWAS (Plissonneau *et al*., 2017).

Whilst multiple developments in effector discovery have increased our understanding of these enigmatic proteins, arguably the explosion in effector research can be attributed to the development of three approaches: genome sequencing, bespoke bioinformatic pipelines, and *Agrobacterium*‐mediated transient expression in planta. Armed with only an annotated genome, even understudied conifer‐infecting fungal pathogens can be screened for the presence of putative effector proteins (Raffaello and Asiegbu, 2017). With this in mind, genome reannotations and improvements to prediction algorithms continuously widen the pool of effector candidates available, especially in well‐studied crop pathogens (Zhong *et al*., 2017; Frantzeskakis *et al*., 2018). Therefore, perhaps the greatest roadblock to effector discovery is the accuracy of genome assembly and annotation, an issue that will take at least 5–10 years to resolve with the inclusion of pangenomes (Cissé and Stajich, 2019).

The genome annotation of multiple isolates through the construction of pathogen pangenomes allows for intraspecific genome analysis and will provide insight into the links between high polymorphisms and host specificity. The use of pangenome analyses has already led to the differentiation between core candidate effectors and novel candidate effectors in *Z. tritici* and *M. oryzae* (Singh *et al*., 2019; Badet *et al*., 2019). Machine‐learning‐based prediction tools as well as the robotic implementation of practical molecular techniques should help to fast track the progress from effector prediction to characterization. This anticipated progress will undoubtedly erode some of the disparity in our interspecies knowledge and lift the veil on the enigmatic filamentous phytopathogen effector repertoire. Many novel functions, locations, interactions, and generic underlying themes remain to be discovered.

## Data Availability

Data sharing is not applicable to this article as no new data were created or analysed in this study.
